# Virtual Interviewing in the Era of COVID-19: A Preliminary Analysis of Otolaryngology Residency Program Costs

**DOI:** 10.1177/2473974X221128908

**Published:** 2022-09-27

**Authors:** Andrew Yousef, Benjamin Bernard, Deborah Watson

**Affiliations:** 1 Department of Otolaryngology–Head and Neck Surgery, University of California San Diego, San Diego, California, USA

**Keywords:** residency interviews, surgical education, virtual interviews, program costs

## Abstract

**Objective:**

A preliminary comparison of the program experience and costs associated with the virtual interview season during the 2020-2021 COVID-19 pandemic against the traditional in-person interview process during the 2019-2020 interview season.

**Study Design:**

Cross-sectional survey.

**Setting:**

Our institutional program launched an online survey via REDCap to otolaryngology programs across the country.

**Methods:**

A 33-item survey was sent to otolaryngology residency program directors regarding their experience and costs associated with virtual interviews during the 2020-2021 cycle and in-person interviews during the previous 2019-2020 cycle. Purchasing cost and opportunity cost were calculated for each program.

**Results:**

Twenty-two programs sent back completed survey responses. Program responses were equally represented among all regions of the United States. In the 2020-2021 interview season, programs received more applications (mean, 400 vs 336 the year prior, *P* < .001) for a similar number of residency spots per program (3.04 in 2020-2021 vs 3.0 2019-2020, *P* = .715). The virtual interview led to more half-day interviews, a shorter duration of each interview, and fewer interviews completed per interview date. Purchasing cost decreased by $1940.46 (73%), and person-hours dedicated to the interview process decreased by 52.36 with the virtual interview. Total savings per program with virtual interviews were an estimated $6941.66.

**Conclusions:**

Virtual interviews in the setting of the COVID-19 pandemic led to a shift in application and interview patterns and was associated with a reduction in costs for programs when compared with the in-person interview format.

The COVID-19 pandemic has transformed many aspects of otolaryngology and medical education. In the clinical arena, the deployment of telemedicine and virtual patient visits has skyrocketed.^1^ In medical education, the entire otolaryngology residency interviewing season was conducted via virtual interviews, an unprecedented shift from the norm of the in-person interviewing format. Virtual interviewing offers advantages and disadvantages to applicants and residency programs. One significant advantage of virtual interviews for applicants is alleviation of the cost burden associated with in-person interviews, which includes the financial aspect and time involvement. Multiple studies have demonstrated how expensive the residency application process is for applicants, with financial costs in excess of $11,500.^2-10^

The in-person residency interviewing process poses significant costs to residency programs as well. Some of the associated costs include lost hospital revenue due to attending physicians’ inability to perform clinical duties on interviewing days, meal costs, and room and equipment rental. While the cost advantages of a virtual interview process from the applicant perspective have been well explored in the literature, there is a paucity of data analyzing the effects of virtual interviewing on residency programs. This study provides a preliminary review of the cost differences with the virtual interview format for otolaryngology residency programs by reviewing survey data collected from a sampling of residency programs throughout the country during the 2019-2020 and 2020-2021 interview cycles.

## Material and Methods

We performed a cross-sectional survey-based study comparing the financial aspects and time involvement of the virtual interview format during the 2020-2021 COVID pandemic and the in-person interviews during the 2019-2020 cycle. This study was submitted to the University of California San Diego’s Institutional Review Board and approved for exemption status.

### Survey Design

A 33-item online survey was designed and sent to otolaryngology residency program directors requesting a comparison of their experience and costs associated with the virtual interview and the in-person interview format (Supplement 1, available online). The survey was modified from a previously published study that focused on the financial costs associated with the surgical residency interview process.^11^ In this previous study, surgical program directors were given a survey to assess program characteristics as well a variety of details related to conducting on-site interviews. The survey was verified and approved by the Association of Program Directors in Surgery’s Research Committee. This survey was then adapted for our study to gather data from the 2020-2021 and 2019-2020 cycles.

### Data Collection

Submitted surveys were collected and managed with REDCap tools hosted at our institution.^12,13^ An invitation to complete the survey was provided to approximately 100 otolaryngology program directors across the country via email. Survey participation was voluntary and anonymous. Email reminders for survey completion were sent 3 and 6 weeks after the initial survey invitation.

Program characteristics were collected, such as type of training program, region, and size of program. Overall application information was then collected: number of applications received each year, number of interview invitations sent, number of interview days, length of interview day, time per interview, and number of interviews per candidate. Program directors were then asked about different components of their interview process, such as whether the interview day included a tour/virtual tour, participation in rounds or conferences, meals, and a social event with the program’s residents.

The program directors were asked to evaluate the financial costs associated with the in-person and virtual interview formats, including meals and snacks, a separate social gathering with food, printing and supplies, room reservations, shuttle tour, venue, and any other fees. Last, the survey focused on the individual opportunity costs or time involvement associated with interviews by program directors, associate program directors, faculty, residency coordinators, residents, and chairs.

### Statistical Analysis

For descriptive statistics, means, medians, and frequencies were calculated as appropriate. We compared the 2020-2021 virtual interview format with the 2019-2020 in-person interview format. Normally distributed continuous data were analyzed with a paired *t* test or analysis of variance and categorical data by a chi-square test.

For the financial cost analysis, purchasing costs were defined as the costs of all goods purchased for interviews. Examples of purchasing costs include funds spent on meals, equipment rentals, and printing supplies, in addition to others. Opportunity cost was defined by costs associated with faculty being removed from clinical duty and other obligations to assist with interviewing of applicants. Opportunity cost was calculated in person-hours, or the number of persons involved in interviewing applicants multiplied by the number of hours worked. Average national wages for chairs, otolaryngology physicians, administrative staff, and residents were used in these calculations.^14^ Stata version 16 (StataCorp) was used for all analysis, and statistical significance was taken at *P* < .05.

## Results

**Figure 1.**
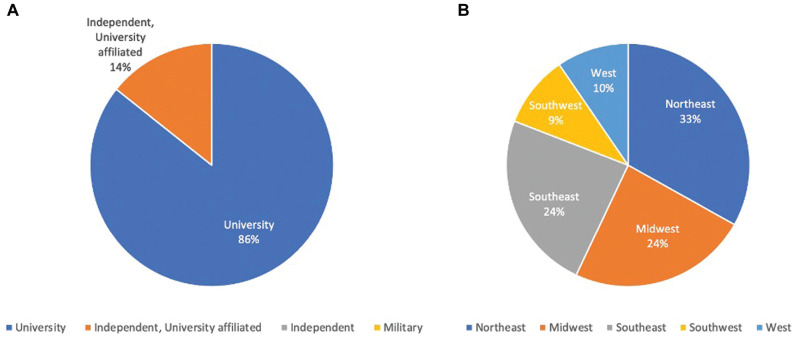
Program demographics: (A) type and (B) region.

A total of 22 programs completed all survey questions for a response rate of 21%. Program type and regions are presented in **Figure 1**. An overall 86.3% of programs that responded were university-based programs, and the remaining were independent programs that were university affiliated. Programs from all regions were represented.

### Applications and Interviews

For the 2020-2021 interview season, programs received a mean 400 applications as opposed to 336 for the year prior (*P* < .001). There was a similar number of residency spots per program, with 3.04 available in the 2020-2021 cycle vs 3.0 in the 2019-2020 cycle (*P* = .715; **Table 1**). Programs sent out a similar number of interview invitations, at means of 53.0 in the 2020-2021 cycle and 51.86 in the 2019-2020 cycle (*P* = .339). Despite similar numbers of invitations, programs completed more total interviews in 2020-2021 (47.23) than 2019-2020 (41.59, *P* = .002). Fewer applicants canceled interviews after accepting the interview offer during the 2020-2021 interview cycle (5.23) as compared with the 2019-2020 cycle (9.09, *P* = .029).

**Table 1.** Effect of the Virtual Interview on Applications and Interviews (22 Programs).^a^

	2019-2020	2020-2021	Absolute difference^b^	*P* value
No. of residents allotted	3 (1.5)	3.04 (1.4)	0.04	.715
Total				
Applications received	336.36 (103.7)	400.0 (110.2)	63.64	<.001^c^
Interview invites	51.86 (20.6)	53.0 (18.6)	1.14	.339
Interviews completed	41.59 (15.2)	47.23 (16.8)	5.64	.002^c^
No. of				
Interview days	2.43 (0.8)	2.89 (1.0)	0.46	.029^c^
Interviews canceled by applicants	9.09 (9.3)	5.23 (5.7)	−3.86	.029^c^
Full day:half day	19:2	13:9	—	.002^c^
Time per interview, min	17.50 (3.0)	16.32 (3.7)	−1.18	.004^c^
No. of interviews/applicant	8.18 (3.0)	7.45 (2.8)	−0.73	.003^c^

a Values are presented as mean (SD).

b 2020/2021 – 2019-2020.

c *P* < .05.

### Interview Components

The virtual interview brought changes to the timing and components of the interview format. An overall 31.8% of programs switched from full-day interviews to half-day interviews (*P* = .002). Programs decreased the number of interviews completed in an interview day from a mean 8.18 to 7.45 (*P* = .003). Additionally, each interview was shorter in duration at a mean 17.5 minutes in 2019-2020 vs 16.3 minutes in 2020-2021 (*P* = .004; **Table 1**). The events related to the interview day also changed with the virtual process. In 2020-2021, 18.2% of programs that offered a tour during in-person interviews did not offer a virtual tour of the hospital. Furthermore, 72.7% programs that provided a meal during in-person interviews did not provide any food vouchers during virtual interviews. Finally, 13.6% of programs did not integrate a resident social event with the virtual process (**Table 2**).

**Table 2.** Interview Components.^a^

	2019-2020	2020-2021
Welcome and introduction	100	100
Virtual tour/tour	95.4	77.2
Participation in rounds/conferences	0	0
Meal provided	90.9	18.2
Resident social	100	86.4

a Values are presented as percentages.

### Cost Analysis

**Figure 2.**
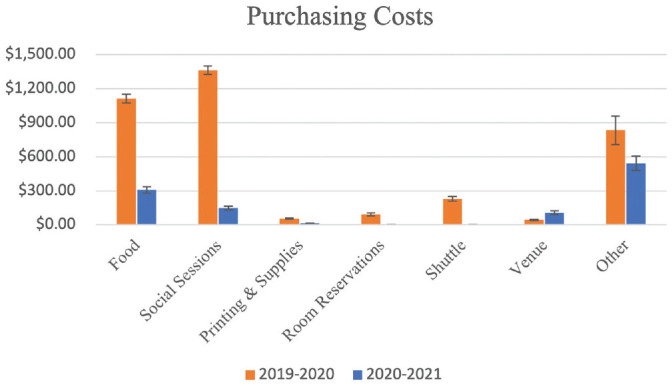
Mean amount spent on purchases between 2020-2021 and 2019-2020. Error bars indicate standard error.

Change in cost for programs with the virtual interview was analyzed. This was divided into 2 categories: purchasing costs and opportunity costs. For purchasing costs, programs spent a mean $1117.06 on food during the 2019-2020 interview cycle as opposed to $288.82 during the 2020-2021 interview cycle (*P* < .001). They spent significantly less on social events at a mean $1329.42 during 2019-2020 vs $137.50 during 2020-2021 (*P* < .001). They also spent significantly less on printing and supplies (*P* = .029) and shuttles (*P* = .039). There were no differences in purchasing costs for room reservations or venue and audiovisual costs (**Table 3**, **Figure 2**). The total purchasing costs dropped by 73.6% from $2637.04 to $696.50 (*P* < .001).

**Table 3.** Purchasing Cost.^a^

	2019-2020	2020-2021	Absolute difference^b^	*P* value
Food	1117.06 (209.35)	288.82 (154.73)	−828.24	<.001^c^
Social sessions	1329.42 (199.08)	137.50 (91.56)	−1191.92	<.001^c^
Printing and supplies	79.64 (29.57)	14.33 (7.68)	−65.31	.029^c^
Room reservations	92.86 (77.55)	0 (0)	−92.86	.148
Shuttle fees	215.63 (110.23)	0 (0)	−215.63	.039^c^
Venues, audiovisual costs	40 (32.34)	100 (93.93)	−60	.259
Total	2637.05 (568.12)	696.59 (319.43)	−1940.46	<.001^c^

a Values are presented in dollars as mean (SE).

b 2020/2021 – 2019-2020.

c *P* < .05.

Opportunity cost was then calculated in person-hours and average cost as described earlier (**Table 4**). In mean total person-hours, there was a 13.8% decrease (52.36) with the virtual interview, though this was not statistically significant. This was similarly seen when examining all personnel involved in the process, with no significant difference in person-hours between 2020-2021 and 2019-2020. Total monetary costs were then calculated from person-hours and purchasing costs. These resulted in a mean $73,216.26 per program for virtual interviews and $80,161.35 per program for in-person interviews, amounting in a total savings of $6941.66 per program with the virtual interview.

**Table 4.** Total Personnel Hours.^a^

	2019-2020	2020-2021	Absolute difference^b^	*P* value
Total	610.44 (360.8)	558.08 (359.3)	−52.36	.286
Program director	52.23 (48.9)	56.64 (47.0)	4.41	.081
Associate program director	47.08 (46.8)	47.92 (45.2)	0.84	.245
Faculty	297.39 (237.8)	257.00 (214.6)	−40.39	.221
Residency coordinator	48.38 (27.0)	48.50 (26.6)	0.12	.307
Department staff	23.67 (20.1)	21.3 (15.4)	−2.37	.50
Residents	118.15 (84.8)	102.71 (84.4)	−15.44	.122
Department chair	23.54 (13.5)	24 (9.8)	0.46	.485
Hospital personnel and others	0 (0)	0 (0)	0	—

a Values are presented as mean (SD) person-hours, calculated as number of people × number of hours.

b 2020/2021 – 2019-2020.

## Discussion

This preliminary study examines the effects of the virtual interview on various aspects of the interview process, including the numbers of applications, components of the interview, and costs associated with this format of residency interviews. It addresses certain methodological differences seen in the current literature and focuses on the program’s perspective rather than the applicant’s perspective with virtual interviews. As a cross-sectional study, the survey allows us to gather information from a sampling of residency programs to understand the changes to program costs associated with virtual interviews in the COVID-19 era.

We found that the advent of the virtual interview led to more applications received per program with more total interviews completed. The virtual interview led to more half-day interviews, shorter duration of each interview, and fewer interviews completed per interview date. There were fewer interviews canceled by applicants. Programs spent less money on food, social sessions, printing, and shuttles, leading to almost $2000 in purchasing supply reductions during virtual interviews. In addition, fewer hours were spent by faculty and staff for the virtual interview process by a calculated average of 52 hours per program.

Conversations related to the costs of residency applications and interviewing are certainly not new. It is well known that residency interviews are very costly for applicants, ranging from $2000 to $15,000 depending on the specialty and study conducted.^3-7,10,11,15-17^ While the cost of interviews for residency programs has not been explored to the same extent as costs to applicants, program costs for residency interviews can reach upward of $100,000.^11^ Given these immense expenses, efforts to reduce the interview budget have been suggested. In 2010, the University of Arizona ophthalmology residency program conducted a prospective study where applicants were offered the choice to interview in person or virtually via video conferencing.^16^ These authors concluded that virtual interviewing is a viable, potentially cost-reducing option for applicants and programs. To reduce program costs further, they suggested conducting interviews in the evenings to limit lost clinical volume and revenue.

The COVID-19 pandemic created a unique situation where altering the interview format was no longer a conversation but rather a necessity. Residency and fellowship programs were forced to implement Zoom, Microsoft Teams, and other video conferencing technologies to conduct their interviews due to the public health crisis. A survey of surgical oncology fellows and faculty showed that 75% of applicants and 100% of faculty stated that the virtual interview process was “very seamless” or “seamless” and cited decreased cost, time saving, and increased efficiency as benefits to virtual interviewing.^18^ While these data support that virtual interviews were well tolerated by applicants and faculty, multi-institutional studies that analyze the cost differences with virtual interviews for residency programs are lacking in our literature. Our study serves as an initial preliminary assessment of these cost differences for our residency programs.

The popularization of the virtual interview due to the COVID-19 pandemic led to multiple changes shown in this study. The virtual interview format led applicants to apply to more programs and cancel fewer interviews. This shift correlates with the decreased time and cost barriers associated with the virtual interview as compared with the in-person format and is consistent with conclusions made by Siddiqui et al.^19^ The travel and accommodation factors are a significant logistical and cost-based barrier otherwise. In addition, programs may have decided to increase the number of interviews to avoid unfilled residency spots as the effect of the virtual interview created much uncertainty. Despite more days of interviewing, the amount of costs on supplies decreased, while the time spent by the program director, associate program directors, faculty, and residents remained relatively unchanged. Costs associated with the virtual interview are not documented well in the literature, but our findings are consistent with Davis et al showing $500 to $1000 of cost savings with the virtual interview process.^20^

The virtual interview led to a change in the interview components, with some programs canceling tours and social sessions and many no longer providing meals to applicants. This transition to a virtual format with less time to interact with faculty and residents in a more relaxed setting can affect a program’s ability to accurately assess a candidate as well as a candidate’s ability to assess a program. In a recently published study out of Wright State University, program directors felt that their ability to determine a candidate’s competitiveness in a virtual format was unchanged, but more subjective components, such as fit for their programs, were more challenging to assess during virtual interviews.^21^ Program directors will need to create strategies that will help them assess the fit of the candidates for their programs.

There are multiple limitations associated with our preliminary study. First, survey results were sent to program directors after the interview season and could be subject to recall bias. Second, we focused our cost analysis strictly on measurable expenses, such as purchasing cost and opportunity cost. However, there are theoretical costs of virtual interviewing as well, such as a possible greater risk for a poor fit between a program and an applicant. This could lead to substantial increased costs for programs over time if the resident transfers out of the program due to a poor fit or disciplinary decisions. This cost is much more challenging to measure but may require programs to incorporate alternative interviewing strategies, such as a hands-on exercise or personality test.^22,23^ Last, our sampling size limits us from having a more elaborate statistical analysis. However, our research provides an initial review of program costs that can help the conversations that are actively occurring in our specialty to ensure successful matches for applicants and programs in a cost-effective manner. A prospective study reviewing all otolaryngology programs’ experiences with the virtual interview format would provide us with the necessary guidance to navigate the changes to our interviewing process.

## Conclusions

During the 2020-2021 virtual interview season, otolaryngology applicants applied to more programs and canceled fewer interviews. Residency programs conducted more interviews that were shorter in duration. Within our sampling of residency programs, virtual interviews created a mean financial cost reduction of $7000 per program, or 8.7% of their total interview costs. The opportunity cost of person-hours was associated with a reduction of $6942, or 13.8% decrease in mean total person-hours. Similar reductive trends in financial and opportunity cost are predicted with a larger study.

## Author Contributions

**Andrew Yousef**, conceptualized the project in addition to leading the data collection and analysis components of the project and helped write the manuscript; **Benjamin Bernard**, assisted with literature review, data analysis, and data visualization and helped write the manuscript; **Deborah Watson**, conceptualized the project, oversaw project administration, assisted with data analysis and revisions of the manuscript.

## Disclosures

**Competing interests:** None.

**Sponsorships:** None.

**Funding source:** None.

## Supplemental Material

sj-pdf-1-opn-10.1177_2473974X221128908 – Supplemental material for Virtual Interviewing in the Era of COVID-19: A Preliminary Analysis of Otolaryngology Residency Program Costs Click here for additional data file. Supplemental material, sj-pdf-1-opn-10.1177_2473974X221128908 for Virtual Interviewing in the Era of COVID-19: A Preliminary Analysis of Otolaryngology Residency Program Costs by Andrew Yousef, Benjamin Bernard and Deborah Watson in OTO Open: The Official Open Access Journal of the American Academy of Otolaryngology-Head and Neck Surgery Foundation
