# Supplementary material for: Virtual Interviewing in the Era of COVID-19: A Preliminary Analysis of Otolaryngology Residency Program Costs
Source: OTO Open. 2022 Sep 27;6(3):2473974X221128908. doi: 10.1177/2473974X221128908 (PMC9520148; doi:10.1177/2473974X221128908)
Supplement: sj-pdf-1-opn-10.1177_2473974X221128908 – Supplemental material for Virtual Interviewing in the Era of COVID-19: A Preliminary Analysis of Otolaryngology Residency Program Costs [file sj-pdf-1-opn-10.1177_2473974X221128908.pdf]

Virtual vs In-Person Interviews for Residency Selection

Dear Program Director,

We are reviewing the format and costs associated with the recruitment, interview, and selection of Otolaryngology residents. We are contacting you because you are a critical constituent in this process.

The following survey will ask you to respond to a number of items about your current screening and interviewing process, estimated number of faculty and staff involved, and other recruitment activities.

Your participation is completely voluntary and your answers will be kept confidential and anonymous.

If you have any questions about the survey or the study, please contact Andrew Yousef, MD at adyousef@health.ucsd.edu .

Thank you for your time,

Andrew Yousef, MD, Resident Surgeon, UCSD Otolaryngology Head & Neck Surgery

Deborah Watson, MD, FACS, Program Director, UCSD Oto-HNS

Which of the following best classifies your training program?

○ University ○ Independent ○ Independent, university affiliated ○ Military ○ Other

---

In which of the following geographical regions is your program located?

○ Northeast ○ Midwest ○ Northwest ○ South ○ East ○ Southwest

What is the estimate of the number of TOTAL applications you received THIS year?

○ 100 - 200 ○ 201 - 300 ○ 301 - 400 ○ 401 - 500 ○ 501 - 600 ○ >600

What is the estimate of the number of TOTAL applications you received LAST year?

○ 100 - 200 ○ 201 - 300 ○ 301 - 400 ○ 401 - 500 ○ 501 - 600 ○ >600

How many residents are you allotted for THIS year? ____________________

How many residents were you allotted for LAST year? ____________________

Approximately how many applicants did you INVITE for interviews THIS year for these positions? ____________________

Approximately how many applicants did you INVITE for interviews LAST year for these positions? ____________________

How many applicants did you interview THIS year? ____________________

How many applicants did you interview LAST year? ____________________

Approximately how many applicants cancelled an interview after accepting it THIS year?

◯ 0 - 5 ◯ 6 - 10 ◯ 11 - 15 ◯ 16 - 20 ◯ 21 - 25 ◯ >25

Approximately how many applicants cancelled an interview after accepting it LAST year?

◯ 0 - 5 ◯ 6 - 10 ◯ 11 - 15 ◯ 16 - 20 ◯ 21 - 25 ◯ >25

Of the applicants you invited to interview, approximately what percentage signaled your program? __________________________________

Of note, this is not your total number of signals nor total percentage of signals. This information should remain confidential. This question is only asking about the applicants invited to interview.

Of the applicants invited for an interview after signaling your program, approximately what percentage cancelled an interview after accepting it? __________________________________

Approximately what percentage of those signaling your program were from out of your region? __________________________________

How many interview days did you have THIS year?

◯ 1 ◯ 2 ◯ 3 ◯ 4 ◯ 5 ◯ 6 ◯ 7 ◯ 8 ◯ 9

How many interview days did you have LAST year?

◯ 1 ◯ 2 ◯ 3 ◯ 4 ◯ 5 ◯ 6 ◯ 7 ◯ 8 ◯ 9

How long was each interview "day" this year?

◯ Half day ◯ Full day ◯ Full day + half day ◯ Two Full days ◯ Other

Please Specify "Other":

____

How long was each interview "day" LAST year

◯ Half day ◯ Full day ◯ Full day + half day ◯ Two Full days ◯ Other

Please specify "Other":

____

Which of the following were components of the interview day THIS year? (Select all that apply)

☐ Program Director (and/or team) Welcome/Introduction ☐ Virtual Tour ☐ Conferences (M&M, Teaching conferences, Grand Rounds, etc.) ☐ Participating in Rounds ☐ Gift card for food ☐ Virtual Social Session with Residents or Faculty ☐ Other

Please Specify "Other"

____

Which of the following were components of the interview day LAST year? (Select all that apply)

☐ Program Director (and/or team) Welcome/Introduction ☐ Tour ☐ Conferences (M&M, Teaching conferences, Grand Rounds, etc.) ☐ Participating in Rounds ☐ Breakfast ☐ Lunch ☐ Dinner ☐ Social Session with Residents or Faculty ☐ Other

Please Specify "Other"

____

Which of the following best describes your interview process THIS year? (Select all that apply)

☐ One-on-one interviews with faculty ☐ Small Group interview ☐ Faculty Panel ☐ Interview with Program Director ☐ Interview with Department Chair ☐ Interview with Residents ☐ Other

Please specify "Other"

____

Which of the following best describes your interview process LAST year? (Select all that apply)

☐ One-on-one interviews with faculty ☐ Small Group interview ☐ Faculty Panel ☐ Interview with Program Director ☐ Interview with Department Chair ☐ Interview with Residents ☐ Other

Please specify "Other"

____

How much time was allocated to each interview THIS year?

◯ 10-15 minutes ◯ 16-20 minutes ◯ 21-25 minutes ◯ 26 minutes or more

How much time was allocated to each interview LAST year?

◯ 10-15 minutes ◯ 16-20 minutes ◯ 21-25 minutes ◯ 26 minutes or more

How many interviews were conducted per applicant THIS year?

○ 1 ○ 2 ○ 3 ○ 4 ○ 5 ○ 6 ○ 7 ○ 8 ○ 9 ○ 10+

How many interviews were conducted per applicant LAST year?

○ 1 ○ 2 ○ 3 ○ 4 ○ 5 ○ 6 ○ 7 ○ 8 ○ 9 ○ 10+

Please provide an approximate dollar amount estimate of how much your program spent during the 2020-2021 and 2019-2020 season on each of the following items for recruitment and interview purposes.

2020-2021 2019-2020
Food (breakfast, lunch, dinner, snacks) ______ ______
Social Sessions/Receptions (i.e. bar tab, snacks) ______ ______
Printing & Supplies ______ ______
Room Reservations ______ ______
Shuttle Fees ______ ______
Tour Guide Fees ______ ______
Venue, A/V costs, etc. ______ ______
Other (please specify): ______ ______

Please provide an approximate dollar amount estimate of how much your program spent during the 2020-2021 and 2019-2020 season on each of the following items for recruitment and interview purposes.

For each of the following groups, please estimate of the average number of hours spent by the following individuals for the 2020-2021 and 2019-2020 recruitment season

Consider all screening, recruitment, preparatory activities, evaluation processes, interview day obligations, and final ranking meetings when making these estimates.

For example, if you have 20 cumulative faculty with the average faculty member spending 5 hours prepping for interviews, 10 hours conducting interviews, and 3 hours in a final group ranking session over the course of interview season. You would put "20" for number of faculty and "18" under average number of hours.

2020-2021 2020-2021 2019-2020 2019-2020
Number of individuals Average Number of Hours Number of individuals Average Number of Hours
Program Director ______ ______ ______ ______
Associate Program Directors ______ ______ ______ ______
Faculty Interviewers ______ ______ ______ ______
Residency Coordinator(s) ______ ______ ______ ______
Department Staff (admins, sim center staff, etc.) ______ ______ ______ ______
Residents ______ ______ ______ ______
Department Chair ______ ______ ______ ______
Hospital Personnel ______ ______ ______ ______
Other ______ ______ ______ ______
